# Multiscale Uncertainty Quantification of Woven Composite Structures by Dual-Correlation Sampling for Stochastic Mechanical Behavior

**DOI:** 10.3390/polym17192648

**Published:** 2025-09-30

**Authors:** Guangmeng Yang, Sinan Xiao, Chi Hou, Xiaopeng Wan, Jing Gong, Dabiao Xia

**Affiliations:** 1 Jihua Laboratory, Foshan 528200, China; yanggm@jihualab.ac.cn; 2 Shien-Ming Wu School of Intelligent Engineering, South China University of Technology, Guangzhou 511442, China; sinanxiao@scut.edu.cn; 3 School of Aeronautics, Northwestern Polytechnical University, Xi’an 710072, China; houchi@nwpu.edu.cn (C.H.); wanxp@nwpu.edu.cn (X.W.); 4 Key Laboratory of Functional Materials and Devices for Informatics of Anhui Educational Institutions, School of Physics and Electronic Engineering, Fuyang Normal University, Fuyang 236037, China

**Keywords:** multiscale simulation, woven composites, uncertainty quantification, multivariate random field, spatial correlation, probabilistic distribution

## Abstract

Woven composite structures are inherently influenced by uncertainties across multiple scales, ranging from constituent material properties to mesoscale geometric variations. These uncertainties give rise to both spatial autocorrelation and cross-correlation among material parameters, resulting in stochastic strength performance and damage morphology at the macroscopic structural level. This study established a comprehensive multiscale uncertainty quantification framework to systematically propagate uncertainties from the microscale to the macroscale. A novel dual-correlation sampling approach, based on multivariate random field (MRF) theory, was proposed to simultaneously capture spatial autocorrelation and cross-correlation with clear physical interpretability. This method enabled a realistic representation of both inter-specimen variability and intra-specimen heterogeneity of material properties. Experimental validation via in-plane tensile tests demonstrated that the proposed approach accurately predicts not only probabilistic mechanical responses but also discrete damage morphology in woven composite structures. In contrast, traditional independent sampling methods exhibited inherent limitations in representing spatially distributed correlations of material properties, leading to inaccurate predictions of stochastic structural behavior. The findings offered valuable insights into structural reliability assessment and risk management in engineering applications.

## 1. Introduction

Woven composites are extensively utilized in the structural design of automotive and aerospace structures owing to their advantages of lightweight, high strength, and ease of integration [1,2,3]. However, due to the complexity of their mesoscopic composition mechanism and the instability of manufacturing processes, woven composites inevitably encounter numerous uncertainties during manufacture. These include fluctuations in weave patterns and variations in material properties and voids [3,4,5]. These characteristics not only result in macroscopic performance variability but also complicate the prediction of damage regions and morphologies. Therefore, it is necessary to develop high-fidelity methods for uncertainty propagation and quantification that fully account for these stochastic factors, thereby enabling more reliable and robust structural design.

Woven composites represent a typical hierarchical structure with inherent uncertainties spanning across micro-, meso-, and macroscales [6,7,8]. During the multi-level propagation of uncertainty corresponding to different stochastic factors, both their marginal probability distributions and the correlation among parameters are required to be clarified. The inverse Nataf transformation [9] is a common method for transforming uncorrelated standard normal variables into correlated variables, though it is primarily suited for cases with linear correlations. For random variables with nonlinear dependencies, Zhou [10] employed a hierarchical computational framework integrating multi-response Gaussian processes and variance-based global sensitivity analysis to effectively propagate uncertainties in simulating the curing manufacturing process of composites. Zhu [11] and Zhang [12] utilized the Copula method to establish the joint distribution of random variables to capture their complex correlations, which was conducted to analyze the influence of random micro-geometric and material property parameters on composite mechanical properties. The performance of this approach relies on the adopted type of copula function [13]. Recently, data-driven methods combined with machine learning have been proposed for multiscale uncertainty quantification and propagation [14,15,16]. By training on a large set of input–output samples, these frameworks do not need to explicitly know the probability distribution of random variables, thereby facilitating the prediction of mechanical properties for woven composites. Nevertheless, the approach often incurs formidable computational costs that remain challenging to overcome. Currently, the uncertainty analysis of mechanical properties of woven composites mainly focuses on how to reduce the computational cost in multiscale analysis by developing theoretical constitutive models [17,18] or surrogate models [19,20,21,22]. However, it is still insufficient to elucidate the correlation between random variables.

The assessment of the strength performance and damage behavior for structures is crucial for reliability and risk management in practical applications. Typically, the spatial distribution of material properties within structures is heterogeneous, leading to randomly distributed localized stress concentrations across different structures. Accurately predicting the stochastic mechanical damage response of structures remains a significant challenge in engineering design. Shah [7] and Tao [20] developed a mesoscale stochastic Representative Volume Element (RVE) model to predict the damage patterns of notched and un-notched composite plates. An independent Monte Carlo sampling technique was employed to assign the randomly simulated material properties to the elements. Wan [6] applied a sampling approach to finite element models at various scales to examine the influence of the randomness of material properties on the damage propagation behavior of composites. However, the sampling method only considered the inter-specimen variability of material properties and neglected their inherent spatial heterogeneity. This limitation would lead to inaccurate prediction of probabilistic mechanical behaviors and discrete damage morphologies of structures compared to reality—an issue that will be further discussed in this paper.

In contrast, the random field (RF) approach [23,24] conceptualizes physical quantities as spatial random fields, enabling a detailed characterization of variation patterns at different locations. Stefanou [25] proposed an RF model based on a Bayesian framework to determine the spatial variability of material properties and investigated the influence of RF parameters on the probabilistic mechanical response of particle-reinforced composites. Arregui-Mena [26] incorporated RF theory within a Monte Carlo framework to generate spatially distributed samples of micro-porosity in nuclear graphite materials, aiming to investigate the impact of pore configurations on the elastic response of graphite. However, the RF models were only applicable to represent the spatial distribution of individual variables and could not characterize cross-correlations among multiple parameters in fiber-reinforced composites.

Multivariate random field (MRF) theory [3,27] provides a framework for modeling the joint spatial distribution of multiple random variables with interdependencies and has recently found preliminary applications in modeling the uncertain mechanical responses of woven composites. Bostanabad [28] utilized a Gaussian MRF to consider the influence of spatial correlations in weave angles, fiber volume fractions, and fiber misalignment angles at various scales on the stochastic stress distribution of cured woven composites. Jiang [29] employed polynomial chaos expansion to construct an MRF that incorporates Copula functions to describe the correlations of spatially distributed uncertain parameters and analyzed the relationship between elastic parameters and the free vibration response of composite plates. Wang [30] established a Vine–Copula-based multivariate cross-correlated non-Gaussian RF to describe the spatial variability of elastic parameters in 2.5D woven SiO_2f_/SiO_2_ composites. The constructed MRF effectively captured the strain fluctuations observed in tensile specimens. These limited studies demonstrated the significant potential of MRF in predicting structural stress/strain distributions and uncertain linear elastic mechanical response. However, there is a pressing challenge in quantifying the cross-correlation and spatial autocorrelation of damage-related random variables within the framework of the MRF to predict the dispersion in strength performance of woven composite structures.

In the present study, a multiscale uncertainty analysis procedure and a novel dual-correlation sampling approach were developed to accurately predict both the probabilistic mechanical performance and the discrete damage morphology of woven composite structures. The rest of this paper is organized as follows: Section 2.1 and Section 2.2 introduce the overall framework of the multiscale uncertainty analysis method and the mechanical testing of plain woven composite specimens. Section 2.3 and Section 2.4 present the analytical models and the methodology for quantifying and propagating uncertainties at each scale, respectively. Section 3 presents the probability distributions of uncertainty sources at various scales and the validation of the multiscale analysis procedure. The focus was placed on the sampling process of the dual-correlation method, the impact on stochastic mechanical responses, and the underlying mechanisms. Concluding remarks and directions for further work are provided in Section 4.

## 2. Materials and Methods

### 2.1. The Framework of Multiscale Uncertainty Analysis

**Figure 1:**
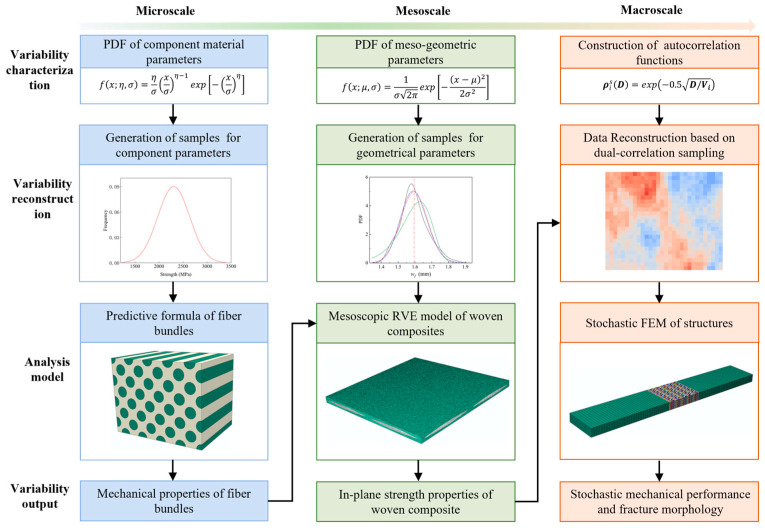
A flow chart of the multiscale uncertainty analysis (Different colors represent different scales.).

The uncertainty analysis of in-plane tensile mechanical properties and damage behaviors in plain woven composites should be performed first. The reinforcement of composites was composed of T300 carbon fibers impregnated with EH301 epoxy resin. Figure 1 illustrates the multiscale uncertainty analysis, including uncertainty propagation from the microscale to the macroscale. The procedure comprises the following key steps:

Identify and input primary random variables at each scale: At the microscale, uncertainties mainly arise from variations in the strength properties of the constituent materials and the fiber volume fraction. At the mesoscale, uncertainties encompass the spatial geometric morphology and material properties of yarns. The geometric characteristics of fiber bundles were identified and collected through Scanning Electron Microscopy (SEM) for the identification and collection of mesoscopic morphological parameters. The statistical distribution functions were determined using probabilistic methods. At the macroscale, the variation in material properties was considered the primary source of uncertainty, which was randomly distributed within different regions of the same component as well as across similar regions of different components. Based on the theory of MRFs, constructing the spatial distribution of material parameters with cross-correlations was crucial for identifying the variability.

Generate samples for random variables: The factors at both the microscopic and mesoscopic scales were treated as a set of input variables, and the Monte Carlo method was employed to generate random samples. At the macroscopic scale, a dual-correlation sampling approach was proposed, where an autocorrelation function was constructed to produce samples with spatial distribution correlations. The samples were further transferred into data coupled with cross-correlations according to the statistical relations of the parameters, which served as input material properties for the Stochastic Finite Element Model (SFEM) of plain woven composite structures.

Incorporate samples across various scales into analysis models: At the microscopic scale, analytical formulas were utilized to predict the stiffness and strength properties of fiber bundles, considering the trade-off between computation efficiency and accuracy. At the mesoscale, based on the geometric parameter samples, the RVE model meshed by tetrahedral elements was generated to predict the in-plane strength properties of plain woven composites. The mesh model enabled high-fidelity geometric modeling of fiber bundles, which is particularly crucial in uncertainty analysis when only minor variations in data are presented. At the macroscale, the centroid coordinates of random elements from the constructed SFEM of tensile specimens were extracted, and material parameters for the elements were generated by using the dual-correlation sampling method. Subsequently, the random characteristics of the load-bearing performance and failure morphology for structural components were numerically simulated and validated by the experimental results.

### 2.2. Mechanical Experiment of Plain Woven Composites

**Figure 2:**
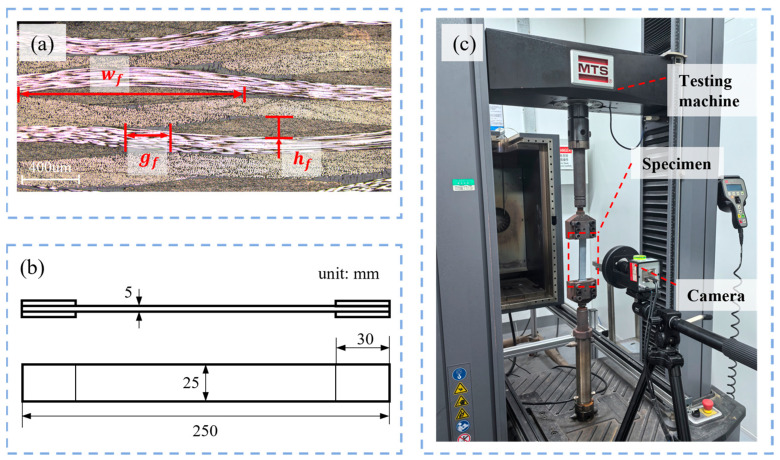
Optical microscopic morphology and mechanical testing for plain woven composites: (**a**) mesoscopic cross-sectional morphology; (**b**) schematic diagram of dimensions for tensile specimens; (**c**) on-site diagram of tensile test.

The mesoscopic morphology of the prepared T300/EH301 plain woven composite was observed using SEM to obtain the statistical data of geometric feature parameters. The composites were fabricated using the vacuum-assisted resin transfer molding (VARTM) technique with a layer-up of [0]_24_ and a fiber volume fraction of 50.0%. Ten observation specimens with dimensions of 40 mm × 10 mm × 5 mm were prepared to generate enough geometric parameter samples. The typical cross-sectional morphology is shown in Figure 2a. The cross-sectional shape and undulation of the fiber bundles could be described, respectively, by the lenticular shape and sinusoidal curve to ensure close contact between the warp and weft fiber bundles. Based on the assumption, three independent geometric parameters, including the width $\left(w_{f}\right)$, thickness $\left(h_{f}\right)$, and gap $\left(g_{f}\right)$, of the fiber bundles were identified to characterize the mesoscopic morphology of the plain woven composites.

Uniaxial tensile tests were conducted on twelve rectangular specimens following standard ASTM D3039 [31]. The geometric dimensions of the specimens and the experimental setup are depicted in Figure 2b,c, respectively. To mitigate stress concentration-induced damage at the clamping regions, aluminum sheets were bonded to both ends of the specimens. The tests were performed using a 100 kN MTS model E45.105 (MTS Systems Co., Ltd., Eden Prairie, MN, USA) universal testing machine under displacement control mode, with a crosshead speed of 12 mm/min (nominal strain rate: 0.001 s^−1^). Full-field strain measurements were acquired via an optical extensometer system based on digital image correlation (DIC). To enable high-resolution optical deformation measurement, a stochastic speckle pattern with high contrast was prepared on the surface of the specimen before mechanical testing. This was achieved by first applying a uniform white matte substrate coating, followed by the deposition of randomly distributed black speckles using a spray-painting technique to ensure high-contrast stochastic features suitable for accurate digital image correlation.

### 2.3. Methodology of Multiscale Modeling

**Figure 3:**
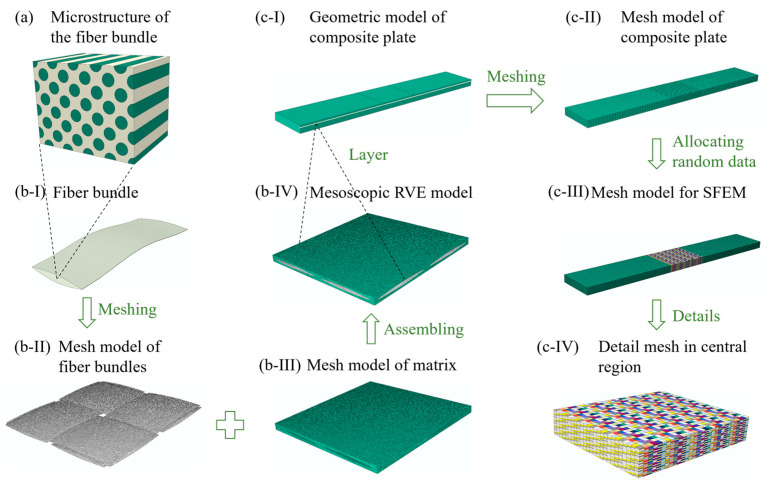
The multiscale modeling approach for plain woven composites: (**a**) the microstructure of fiber bundles (Green: fiber filament and white: matrix); (**b-I**) the geometric configuration of a single-fiber bundle; (**b-II**) the mesh model of orthogonally woven fiber bundles; (**b-III**) the mesh model of complementary matrix; (**b-IV**) the assembling RVE mesh model; (**c-I**) the geometric model of tensile specimens; (**c-II**) the initial macroscopic mesh model of tensile specimens; (**c-III**) the SFEM with randomly assigned material parameters; (**c-IV**) the detailed mesh in the central region (Different colors represent different material parameters.).

The multiscale modeling process for plain woven composites was depicted in Figure 3. Figure 3a shows the microstructure of a fiber bundle, wherein the fiber filaments are assumed to be uniformly distributed (Figure 3b-I) with a well-bonded interface with the matrix. At the mesoscale, plain woven composites consist of orthogonally woven fiber bundles (Figure 3b-II) and the complementary matrix (Figure 3b-III). Considering the periodic features of mesoscopic morphologies, the RVE model (Figure 3b-IV), divided by tetrahedral elements, was utilized to predict the mechanical properties of composites. At the macroscale, tensile specimens were constructed by stacking multiple layers of woven composites at specified ply angles (Figure 3c-I). Consequently, the geometrical model was meshed using regular hexahedral elements, with each layer being divided into a single layer of elements (Figure 3c-II). The central region was refined to assign randomly generated material parameters (Figure 3c-IV). Ultimately, the SFEM (Figure 3c-III) was established to calculate the mechanical response and failure morphology of the laminate structure.

#### 2.3.1. The Microscopic Analytical Model for Fiber Bundles

At the microscale, the fiber bundles exhibited transversely isotropic mechanical behaviors. To balance accuracy and computational cost, the widely used Chamis analytical formula [32] was employed to predict the stiffness of the fiber bundle. Simultaneously, the strength parameters were calculated using the formula in Reference [33], which is expressed as

(1) $$\left\{\begin{array}{c} X_{1t}^{y}=V_{f}^{y}X_{ft}\\ \begin{array}{c} X_{1c}^{y}=V_{f}^{y}X_{fc}\\ \begin{array}{c} X_{2t}^{y}=X_{mt}\left(1-\sqrt{V_{f}^{y}}-V_{f}^{y}\right)\left(1-\frac{E_{m}}{E_{f,22}}\right)\\ X_{2c}^{y}=X_{mc}\left(1-\sqrt{V_{f}^{y}}-V_{f}^{y}\right)\left(1-\frac{E_{m}}{E_{f,22}}\right) \end{array}\\ S_{13}^{y}=X_{ms}\left(1-\sqrt{V_{f}^{y}}-V_{f}^{y}\right)\left(1-\frac{G_{m}}{G_{f,12}}\right) \end{array}\\ S_{23}^{y}=X_{ms}\frac{1-\sqrt{V_{f}^{y}}\left(1-\frac{G_{m}}{G_{f,23}}\right)}{1-\sqrt{V_{f}^{y}}\left(1-\frac{G_{m}}{G_{f,23}}\right)} \end{array}\right.$$

where $V_{f}^{y}$ is the fill coefficient of fiber bundles calculated based on the fiber volume fraction and the fiber bundle volume fraction. $X_{1t}^{y}$, $X_{1c}^{y}$, $X_{2t}^{y}$, $X_{2c}^{y}$, $S_{13}^{y}$, and $S_{23}^{y}$ represent the strength of fiber bundles, with subscript numbers indicating the directions and *t* and *c* referring to tension and compression. $E_{f,22}$, $G_{f,12}$, and $G_{f,23}$ are stiffness parameters. $X_{ft}\;$ and $X_{fc}$ represent the fiber filament strengths. $E_{m}$ and $G_{m}$ are the moduli of the matrix, while $X_{mt}$, $X_{mc}$, and $X_{ms}$ are the tensile, compressive, and shear strengths of the matrix.

#### 2.3.2. The Mesoscopic RVE Model for Plain Woven Composites

At the mesoscale, plain woven composites were composed of periodically interlaced fiber bundles embedded in a surrounding matrix. An RVE model with periodic boundary conditions was employed to characterize the intricate mesoscale geometric features of composites. Three independent geometric parameters $w_{f}$, $g_{f}$, and $h_{f}$, were identified to establish the geometric RVE model, where the sinusoidal undulation curve of fiber bundles is mathematically described by

(2) $$y_{1}\left(x\right)=\frac{h_{f}}{2}\left[1-\cos\left(\frac{\pi x}{u_{f}}\right)\right]$$

and the lenticular cross-section of fiber bundles is described by

(3) $$y_{2}\left(x\right)=-h_{f}\cos\left[\beta \left(x-u_{f}\right)\right]$$

where

(4) $$\beta =\frac{2}{w_{f}}arc\cos\left(\sin^{2}\frac{\pi w_{f}}{4\left(u_{f}\right)}\right),{\;\;\;\;\;\;\;\;\;u}_{f}=g_{f}+w_{f}$$

In uncertainty analysis, the RVE model is repetitively constructed based on randomly generated samples of geometric parameters. Since the sample data may exhibit minor variations, this is particularly prominent for the flattened fiber bundles with a small ratio of $g_{f}/w_{f}$ and $h_{f/w_{f}}$ in this study. Therefore, the three-dimensional four-node tetrahedral elements C3D4, which offered better adaptability compared to the pixel-based hexahedral elements [34], were used for mesh generation to achieve high-fidelity modeling in both the dimensional accuracy and surface characteristics of the fiber bundles. The irregularity of tetrahedral elements introduced difficulties in defining the main material directions and periodic boundary conditions for the RVE model. A detailed solution to this issue is provided in Appendix A. In addition, the damage constitutive relationships for the fiber bundles and matrix based on continuum mechanics are also introduced in Appendix A.

#### 2.3.3. The Macroscopic SFEM for Composite Components

At the macroscale, the in-plane tensile specimen with a stacking sequence of [0]_24_ was taken as the target macroscopic composite structure. The model was meshed with C3D8 elements with a global seed size of 2.0 mm. In order to capture the discrete damage morphology, the performance degradation of elements within the central region (30 mm × 25 mm) was considered, where random strength data were allocated, while the remaining elements were assigned solely elastic constants in the SFEM. The strength data was generated based on the probability distribution obtained from mesoscale RVE models and the dual-correlation sampling approach proposed in this study. A total of 18,000 elements were divided in the random region with an elemental size of 1.0 mm × 1.0 mm × 0.21 mm to achieve convergent prediction results.

Since the tensile strength of laminates was deemed equivalent to that of the material for the identical ply sequence, the maximum stress criterion was adopted as the damage criterion for tensile specimens. The complete displacement constraint was applied at one end, and the longitudinal displacement was applied at the opposite end. The simulation analysis of the SFEM was performed using the Abaqus/Standard nonlinear module.

### 2.4. Correlation Quantification and Propagation Model

The above multiscale model provides a solid foundation for analyzing structural damage behavior, and the key to conducting uncertainty analysis on it is to accurately propagate the cross-correlation between different material properties and quantify the spatial autocorrelation of material properties within structures. In this paper, the Pearson correlation analysis method [35] was first utilized to quantify the correlation degree among the material parameters predicted by the micro-to-mesoscale model. Subsequently, a novel dual-correlation sampling method based on the theory of MRFs was proposed. The method ensures that the material parameters assigned to the elements of the macroscopic SFEM embody both cross-correlation and spatial autocorrelation, thereby achieving the quantitative analysis of the discrete distribution of structural mechanical properties and damage morphologies simultaneously.

#### 2.4.1. The Correlation Analysis of Material Parameters

The Pearson correlation coefficient method is a commonly used analytical approach to measure the correlation between a random variable ***X*** and another random variable ***Y***, expressed as

(5) $$r=\frac{V\left(Y,X\right)}{\sigma_{Y}\sigma_{X}}$$

where $\sigma_{X}$ and $\sigma_{Y}$ represent the standard deviations of ***X*** and ***Y***, respectively. $V\left(Y,X\right)$ is the covariance between ***Y*** and ***X***. The Pearson correlation coefficient *r* is a statistical measure used to quantify the degree of linear correlation, with its value ranging from −1 to 1. A value close to 1 implies strong positive correlation, a value near −1 indicates strong negative correlation, and a value near 0 means no linear correlation.

Noting that the Pearson method is suitable for measuring linear correlations between variables, a distance correlation analysis method [36] capable of measuring more general nonlinear correlations was proposed to verify the effectiveness of this approach in assessing the correlations among material properties of plain woven composites. The distance correlation coefficient R ranges between 0 and 1, where a value close to 0 indicates no correlation, and a value close to 1 signifies a strong correlation. However, it cannot reflect positive or negative correlation. Similar to the Pearson correlation coefficient, the R is defined as

(6) $$R=\frac{V\left(X,Y\right)}{\sqrt{V\left(X\right)V\left(Y\right)}}\;V\left(X\right)V\left(Y\right)>0$$

where $V\left(X\right)$ and $V\left(Y\right)$ are the distance variances of X and Y. The distance covariance $V\left(X,Y\right)$ can be estimated as

(7) $$V\left(X,Y\right)=\sqrt{\frac{1}{n^{2}}\sum_{k,l=1}^{n}A_{kl}B_{kl}}$$

and

(8) $$\left\{\begin{array}{c} A_{kl}=a_{kl}-a_{k\cdot }-a_{\cdot l}+{\overset{\text{-}}{a}}_{\cdot \cdot }\\ \begin{array}{c} a_{kl}=\left\|x_{k}-x_{l}\right\|\\ a_{k\cdot }=\left[\underset{n}{\underbrace{{\overset{\text{-}}{a}}_{k\cdot },{\overset{\text{-}}{a}}_{k\cdot },\ldots {\overset{\text{-}}{a}}_{k\cdot }}}\right],a_{l}=\left[\underset{n}{\underbrace{{\overset{\text{-}}{a}}_{\cdot l},{\overset{\text{-}}{a}}_{\cdot l},\ldots {\overset{\text{-}}{a}}_{\cdot l}}}\right] \end{array}\\ {\overset{\text{-}}{a}}_{k\cdot }=\frac{1}{n}\sum_{l=1}^{n}a_{kl},\;{\overset{\text{-}}{a}}_{\cdot l}=\frac{1}{n}\sum_{k=1}^{n}a_{kl},\;{\overset{\text{-}}{a}}_{\cdot \cdot }=\frac{1}{n^{2}}\sum_{k,l=1}^{n}a_{kl} \end{array}\right.$$

where *n* is the sample number, and $x_{k}$ and $x_{l}$ are the samples of variable ***X***.

#### 2.4.2. The Correlated Sampling Method for the SFEM

The dual-correlation sampling was developed from the theory of MRFs. For the material parameter vector, $X={\left(X_{1},X_{2},\ldots ,X_{p}\right)}^{T}\in R^{p}$, which follows a multivariate normal distribution:

(9) $$X\left(s\right)\sim Ν\left(\mu ,\;\Sigma \right)\;$$

The covariance matrix C can be decomposed as

(10) $$C=C^{s}⨂C^{m}$$

where $C^{s}$ is the spatial autocorrelation covariance matrix constructed from the autocorrelation function; and $C^{m}$ is the cross-correlation covariance matrix constructed from the Pearson correlation coefficient matrix. ⨂ denotes the Kronecker product, and according to its operational properties,

(11) $$C^{s}⨂C^{m}=\left(L^{s}{\left(L^{s}\right)}^{T}\right)⨂\left(L^{m}{\left(L^{m}\right)}^{T}\right)=\left(L^{s}⨂\;L^{m}\right){\left(L^{s}⨂\;L^{m}\right)}^{T}$$

The order of Cholesky decomposition for $L^{s}$ and $L^{m}$ is exchangeable. The final sample, coupling with spatial autocorrelation and cross-correlation, is expressed as

(12) $$Y^{s,m}=\mu +\left(L^{s}⨂\;L^{m}\right)Z,\;\;\;Z\sim N\left(0,I\right)$$

**Figure 4:**
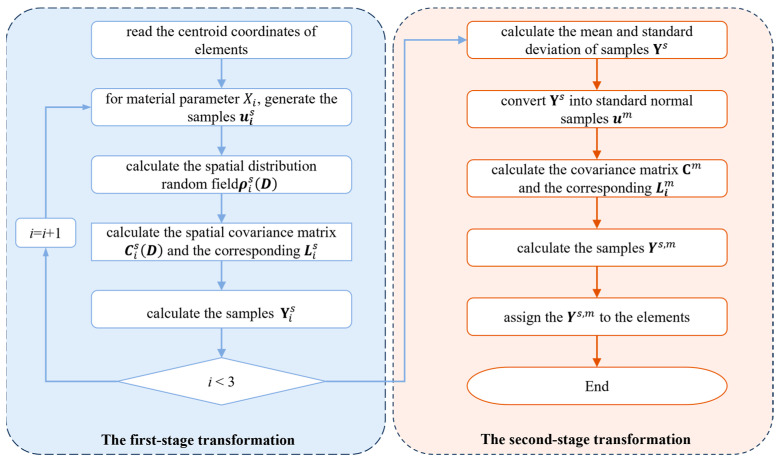
A flow chart of the dual-correlation sampling method.

The detailed dual correlation sampling process is depicted in Figure 4. Firstly, a spatial autocorrelation function is constructed to convert standard normally distributed random samples into spatially autocorrelated samples along the spatial dimension. Then, based on the principle of equal probability [37], the samples are further converted into cross-correlated material data in material dimensions. The specific process of dual-correlation sampling is delineated as follows:

(1) For the spatial centroid coordinates $T\in R^{q\times 3}$ in the random region of the SFEM, where *q* is the number of random elements. For the variable $X_{i}$, an exponential autocorrelation function is selected to construct the spatial distribution random field, expressed as
  (13) $$\rho_{i}^{s}\left(D\right)=exp\left(-0.5\sqrt{D/V_{i}}\right)$$
  where $\rho_{i}^{s}$ represents the spatial correlation coefficient matrix for $X_{i}$, and $D\in R^{q\times q}$ is the Euclidean distance matrix among all random elements. $V\in R^{p\times 3}$ is the autocorrelation distance within the three-dimensional spatial domain, which defines the degree of spatial variability of the random field. A smaller value of ***V*** signifies weaker spatial correlation, and as the value approaches 0, the random field degrades into spatially independent random variables [28]. Conversely, a larger value of ***V*** indicates strong spatial correlation, and as the value tends to infinity, the samples converge into a set of deterministic values.
(2) For the variable $X_{i}$, the calculation of spatial covariance matrix $C_{i}^{s}$ is performed as
  (14) $$C_{i}^{s}\left(D\right)=\sigma_{i}^{2}\rho_{i}^{s}\left(D\right)$$
  where $\sigma_{i}$ is the standard deviation of $X_{i}$. Then, Cholesky decomposition is conducted for the covariance matrix given as
  (15) $$C_{i}^{s}\left(D\right)=L_{i}^{s}{\left(L_{i}^{s}\right)}^{T}$$
(3) Generate a set of random samples $u_{i}^{s}$ that follow the standard normal distribution and calculate the parameter $Y_{i}^{s}$ according to Equation (16), where $\mu_{i}^{s}$ is the mean of *X**_i_***:
  (16) $$Y_{i}^{s}=\mu_{i}^{s}+L_{i}^{s}u_{i}^{s}$$
(4) Repeat the above steps for each variable *X_i_* to obtain the samples $Y^{s}$. After the first-stage transformation, the $Y^{s}\in R^{p\times q}$ possesses spatial autocorrelation but lacks cross-correlation.
(5) A similar procedure is carried out to further transform the variable $Y^{s}$. First, the probability distribution of samples $Y^{s}$, including the mean $\mu^{m}$$\in R^{p\times 1}$ and standard deviation $\sigma^{m}$, is calculated. By incorporating the correlations ρ obtained from the correlation analysis in Section 2.4.1, the covariance matrix $C^{m}$ is calculated as
  (17) $$C^{m}=\sigma^{m}\rho {\left(\sigma^{m}\right)}^{T}$$
(6) Then, Cholesky decomposition on $C^{m}$ is performed to obtain the lower triangular matrix $L^{m}$. Convert $Y^{s}$ into standard normal distributed samples $u^{m}$ based on the $\mu^{m}$ and $\sigma^{m}$. Finally, the samples $Y^{s,m}$ that incorporate both cross-correlation and spatial autocorrelations are obtained:
  (18) $$Y^{s,m}=\mu^{m}+L^{m}u^{m}$$

## 3. Results

### 3.1. The Probability Distribution of Input Variables

The random input variables used to calculate the probability distribution of material properties at the micro-to-mesoscale were determined in this section. Among them, the random microscopic constituent materials’ parameters were adopted to compute the mechanical properties of the fiber bundles using Equation (1). Subsequently, the calculated mechanical properties were incorporated into the mesoscopic RVE model with the sampled meso-geometric parameters to statistically obtain the probability distribution of the in-plane strength for plain woven composites.

#### 3.1.1. The Probability Distribution of Microscopic Constituent Material Parameters

This paper considers the randomness of constituent material parameters at the microscale. Through preliminary parametric analysis, it has been determined that four strength parameters, along with the fiber volume fraction, have a significant impact on the in-plane strength properties of the T300/EH301 plain woven composite. Therefore, at the microscale, the five parameters were assumed to follow a Weibull function and were selected as listed in Table 1. Since the coefficients of variation (*CoV*.) of $X_{fc}$, $X_{mc}$, and $V_{f}$ were not provided in the literature, they were set to 5.00% [38,39].

#### 3.1.2. The Probability Distribution of Mesoscopic Geometric Parameters

**Figure 5:**
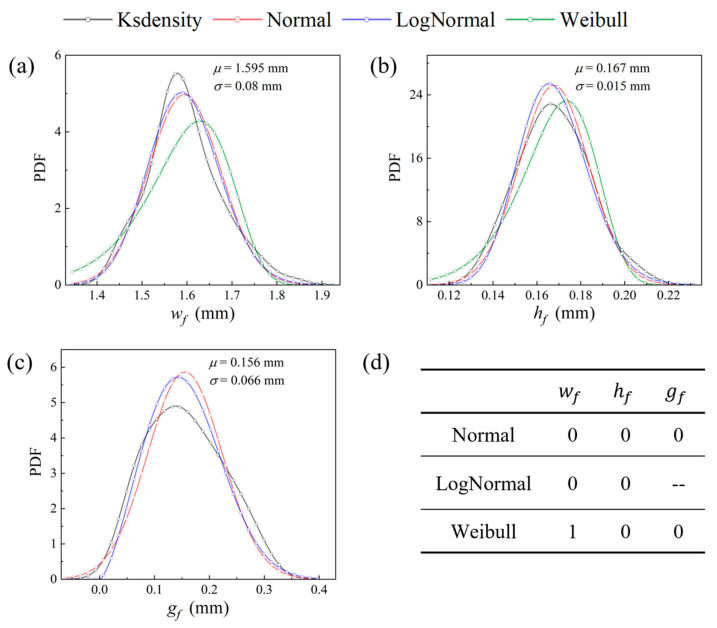
The probability distribution of the mesoscopic geometric parameters: (**a**) $w_{f}$; (**b**) $h_{f}$; (**c**) $g_{f}$; (**d**) results of hypothesis testing. Acceptance: 0 and rejection: 1.

**Figure 6:**
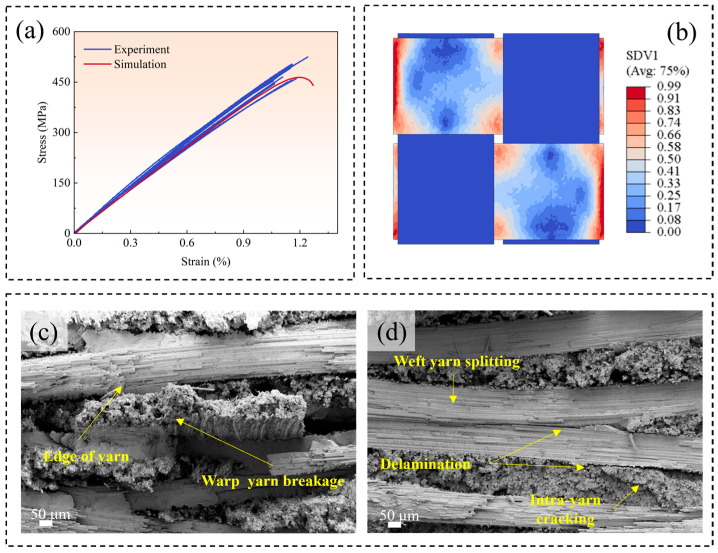
A comparison of the stress–strain curves and damage morphologies obtained from the RVE model and the experiment: (**a**) a comparison of the stress–strain curves obtained from the experiment and the RVE model; (**b**) the damage contour pattern of the warped fiber bundle predicted by simulation, showing that fractures occur in the intersection area of fiber bundles; (**c**,**d**) the fracture morphologies of composites observed by SEM, showing that the breakage of warp fibers bundles occurs in the edge regions of weft fiber bundles.

At the mesoscale, the randomness of three meso-geometric parameters was considered, and hypothesis testing methods [40] were employed to determine their probability distribution functions (PDFs). The PDFs based on Kernel density estimation [41] for the data gathered using an optical microscope are plotted in Figure 5. It can be observed that the coefficients of variation for $w_{f}$ and $h_{f}$ are within 10%, whereas, attributed to nesting and dislocation phenomena [42] during the preparation process, the gap of fiber bundles $g_{f}$ exhibits notable dispersion. Three statistical distribution functions were selected for hypothesis testing on the acquired data: normal distribution, lognormal distribution, and Weibull distribution. Specifically, normal distribution was assessed using the Lilliefors test due to its high calibration efficiency, while lognormal distribution and Weibull distribution were evaluated using the highly versatile Anderson-Darling test [43]. At the significance level of 0.05, the hypothesis testing results are summarized in Figure 6d, where 0 and 1 indicate acceptance and rejection of the hypothesis, respectively. Notably, the lognormal distribution function is not applicable for testing the data of $g_{f}$ due to the negative portion of the PDFs.

The results indicate that all three meso-geometric parameters follow the normal distribution function with sufficient sample capacity. Consequently, normal distribution was chosen as the statistical function for these geometric parameters, with the distribution parameters shown in Figure 5.

### 3.2. The Results of Mesoscopic Analysis

In this section, the validation of the mesoscopic RVE model in predicting the in-plane strength of composites was first verified by comparing with experimental results. On this basis, random samples were extracted to conduct the uncertainty analysis of material properties. Through probabilistic analysis, the PDFs and cross-correlation of the three in-plane strengths were obtained, which provided a sampling basis for the material parameters used in the subsequent uncertainty analysis of structures. Additionally, the dominant factors influencing the in-plane strength of composites were analyzed.

The comparison of in-plane tensile stress–strain curves obtained from the experiment and RVE model is represented in Figure 6a. The curve predicted by the RVE model falls within the range of the experimental curves. Furthermore, Figure 6b illustrates the warp-direction damage state of plain woven composites at the peak load. As the warp fiber bundles serve as the primary loading-bearing components under in-plane tensile loading, it can be observed that the damage predominantly occurs in the interlacing regions of fiber bundles, ultimately leading to the formation of a fracture band nearly perpendicular to the load direction. For comparison, Figure 6c,d presents the fracture damage morphology of a typical composite specimen by SEM observation. Various damage forms are observed via SEM, including the fracture and splitting of warp and weft fiber bundles, as well as interlaminar and intralaminar delamination due to the misalignment and nesting phenomenon. However, there is a fracture band at the edge of the weft fiber bundles, where the entire warp fiber bundles have fractured. The observation is consistent with the prediction of the RVE model, indicating that the RVE model can predict the damage characteristics of plain woven composites.

**Figure 7:**
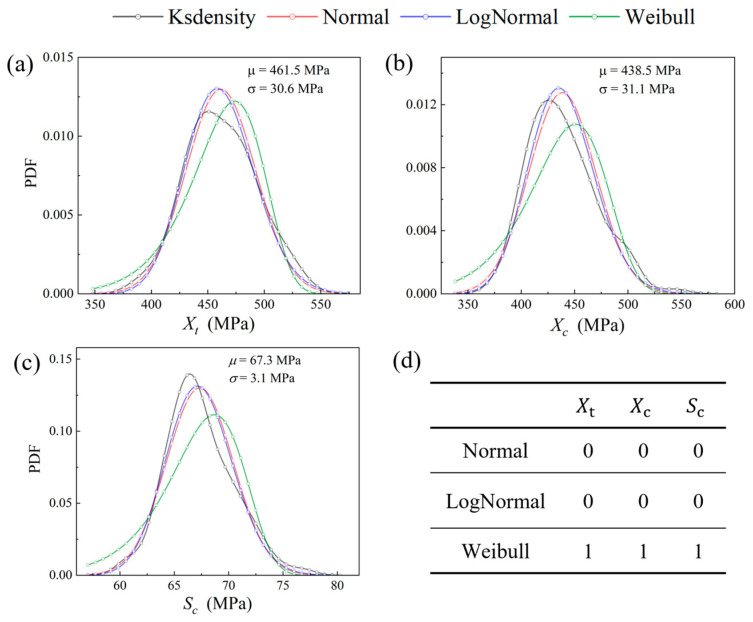
Probability distribution and curve fitting of in-plane strength parameters of plain woven composites: (**a**) tension strength $X_{t}$; (**b**) compression strength $X_{c}$; (**c**) shear strength *S_c_*; (**d**) results of hypothesis testing. Acceptance: 0 and rejection: 1.

Subsequently, 200 sets of samples of micro- and mesoscopic random factors were extracted for conducting uncertainty analysis of composites. The PDFs of the in-plane strength parameters, including tensile strength $X_{t}$, compressive strength $X_{c}$ and shear strength $S_{c}$ for composites, are illustrated in Figure 7. In the context of the multiscale propagation of randomness, it is essential to accurately describe the probability distribution and correlations among variables. Therefore, hypothesis testing on the collected data was first applied by using three statistical distribution functions: normal distribution, lognormal distribution, and Weibull distribution. The results indicate that, at a significance level of 0.05, all three in-plane strength parameters of the composites are consistent with both normal and lognormal distributions, while none of the parameters conform to the Weibull distribution. Based on these findings, normal distribution was selected to describe the material strength parameters.

Furthermore, the correlation among three strength parameters of composites was analyzed using the correlation methods, with the results listed in Table 2. Both methods yield correlation coefficients with absolute values ranging from 0 to 1.0; values closer to 1.0 indicate stronger correlations. It is evident that the Pearson method unanimously reveals a strong correlation among mechanical parameters, especially for the parameters $X_{t}$ and $X_{c}$, which underscores the necessity of incorporating the correlations into structural analysis to achieve a more precise assessment of mechanical behaviors. Notably, the two methods yield identical correlation ranking order for the strength parameters of the composite, suggesting that the Pearson method effectively measures the correlation among the in-plane strength parameters of the plain woven composite. Therefore, the result of the cross-correlation of strength parameters based on the Pearson method was employed in the subsequent correlation sampling analysis for macroscopic SFEMs.

The results of correlation analysis also reveal the mechanism underlying the normal distribution of the three strength parameters of woven composites. The strengths are derived from the same set of micro- and mesoscale random variables through the multiscale model. Given that the uncertainty variables follow a normal distribution, the strength outputs, as weighted combinations of multiple random variables in the multiscale model, naturally tend toward a normal distribution according to the central limit theorem.

**Figure 8:**
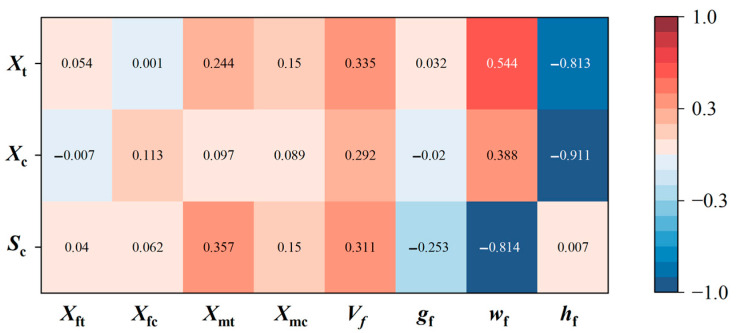
Sensitivity analysis results of in-plane strength parameters for plain woven composites to random input variables of constituent material properties and mesoscopic geometric parameters.

Finally, Figure 8 presents the sensitivity analysis results of material strengths to random input factors to reveal the underlying mechanisms of the statistical correlation using the Pearson method. Compared to the random factors of constituent materials, the strength properties are more sensitive to variations in mesoscopic geometric morphologies. Specifically, $X_{t}$ and $X_{c}$ are jointly influenced by the $w_{f}$ and $h_{f}$ of fiber bundles with similar trends, thereby exhibiting significant positive correlation. However, the parameter *S_c_* is related to the fiber volume fraction $V_{f}$ and matrix properties except $w_{f}$, resulting in weak correlation with the other two strength parameters. It is worth emphasizing that the sensitivity analysis results provide a valuable perspective on enhancing the mechanical properties of plain woven composites. Since the ratio of $w_{f}$ and $h_{f}$ exhibits the highest sensitivity to material properties, it can effectively reduce the fluctuation ratio of fiber bundles by increasing the compaction force during the prepreg preparation process or adopting thin-ply composites [44], thereby leading to a significant enhancement in the in-plane strength performance of the plain woven composites.

### 3.3. The Results of Macroscopic Analysis

Based on the probability distributions of the in-plane strength parameters of composites, dual-correlation sampling was employed to assign the parameters with both cross-correlation and spatial autocorrelation to the SFEM of the tensile specimen. Additionally, the physical significance of the sampling method was investigated. Subsequently, the SFEMs with various spatial autocorrelation distance coefficients ***V*** were established to conduct a comprehensive study on the influence of the spatial distribution of material parameters on the predicted stochastic mechanical properties and damage patterns of macroscopic structures. This analysis provided guidance for structural reliability analysis.

#### 3.3.1. Analysis of Samples Obtained Through Dual-Correlation Sampling

**Figure 9:**
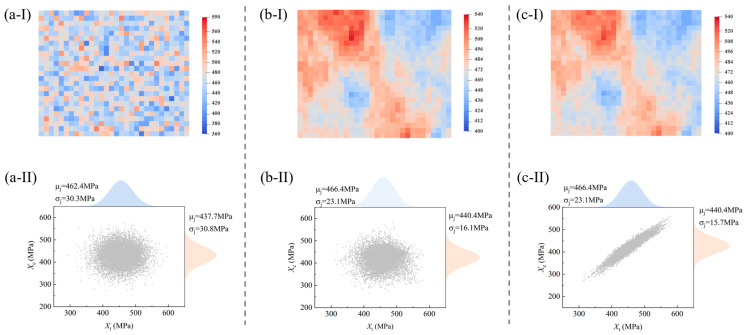
The dual-correlation sampling process of material parameters, including distribution pattern via (**a-I**) independent random sampling, (**b-I**) spatial correlation sampling, and (**c-I**) dual-correlation sampling and data scatter plots, and marginal distribution via (**a-II**) independent random sampling, (**b-II**) spatial correlation sampling, and (**c-II**) dual-correlation sampling.

Figure 9 illustrates the dual-correlation sampling process for the macroscopic tensile specimen. Specifically, Figure 9a presents the samples of *X_t_* obtained through the completely independent sampling method, Figure 9b displays the samples via a single application of the spatial autocorrelation sampling method, and Figure 9c exhibits the samples derived from the dual-correlation sampling method. The series of subplots (I) presents the distribution contour patterns of *X_t_* assigned to the random regions of SFEMs, and the series of subplots (II) depicts the data scatter plots and marginal distribution plots between *X_t_* and *X_c_*.

In the initial stage of the sampling process, the strength parameters are randomly and independently allocated to the elements of SFEMs. Consequently, the contour map of *X_t_* exhibits a completely random, scattered distribution (Figure 9a-I), with no correlation between parameters (Figure 9a-II). After the first-stage spatial autocorrelation transformation (***V*** = 100·E, where $E\in R^{3\times 3}$, with all elements being 1.0), the values of strength parameters display a blocky distribution within the structure, indicating that their spatial distribution is influenced by the properties of surrounding materials. Meanwhile, there remains independence between the strength parameters *X_t_* and *X_c_*. Following the second-stage transformation of dual correlation, a “weakened zone clustering and strong zone continuous” distribution appears, where the parameter *X_t_* not only exhibits a regular distribution pattern but also forms a strong correlation with *X_c_*. The correlation coefficient calculated by the Pearson method is consistent with the result in Table 2, which demonstrates the capability of the dual-correlation sampling method to retain the correlation information between material properties. It should be noted that the second-stage transformation is based on the samples of *X_t_* to perform cross-correlation transformation on other parameters, including *X_c_* and *S_c_*. Therefore, the probability distribution of *X_t_* remains unchanged, while the probability of *X_c_* undergoes alterations. Ultimately, the dual-correlation sampling methodology achieves the allocation of random material samples in SFEMs that incorporate both cross-correlation and spatial autocorrelation.

It should be noted that the probability distribution (denoted as $f_{j}$) of the material parameters in the series of subplots (II) differs from the pre-sampling distribution $f_{i}$ in Figure 7. Physically, there is no inherent connection between the two types of probability distribution, and the distinction is summarized in Table 3. From the perspective of physical significance, the dual-correlation sampling reflects that the inter-specimen variability of material properties matches the distribution $f_{i}$, while the intra-specimen heterogeneity follows the distribution $f_{j}$. This situation is more accurately corresponding to the actual distribution of material properties in structural components, which is further clarified in subsequent analyses.

#### 3.3.2. Analysis of Stochastic Mechanical Response of Structures Predicted by SFEMs

To comprehensively analyze the influence of the spatial distribution of material parameters on the probabilistic mechanical properties and the discrete damage morphologies of structures, SFEMs with various parameter values of ***V*** were established to predict structural damage behavior, as shown in Table 4. For comparison, the independent sampling model (*i*-SFEM) and the deterministic finite element model (*d*-FEM) were also developed. The input parameters for the *d*-FEM are the mean values of the in-plane strength parameters of materials, as depicted in Figure 8.

**Figure 10:**
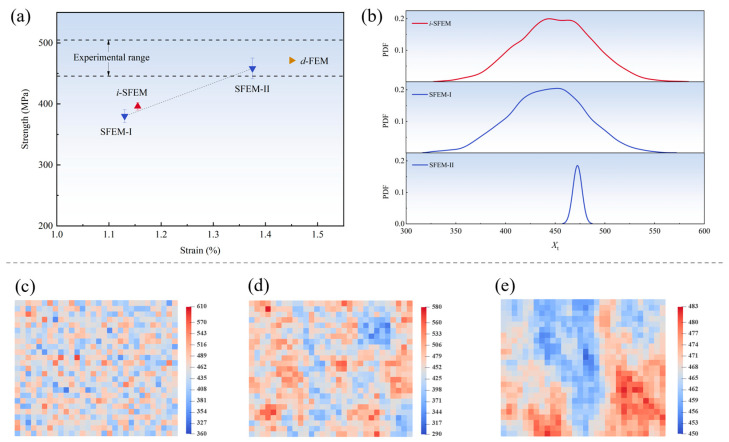
The predicted strength of tensile specimens and sample distribution for three SFEMs: (**a**) comparison of the strength and error bars of tensile specimens obtained from the three SFEMs, *d*-FEM, and experiments; (**b**) the statistical probability distribution of samples for three SFEMs; (**c**) the sample distribution of the *i*-SFEM; (**d**) the sample distribution of SFEM-I; (**e**) the sample distribution of SFEM-II.

Each set of SFEMs comprises 20 sets of random samples, and the predicted strength of tensile specimens and sample distribution are presented in Figure 10. It can be observed that the predicted mean value based on the *i*-SFEM is significantly lower than both the experimental range and the result from the *d*-FEM. The discovery seems intriguing as it appears to contradict the conclusion in References [7,20] where the predicted result from the *i*-SFEM is lower than that from the *d*-FEM, while showing good consistency with experimental curves. In fact, both this study and References [7,20] demonstrate that the results of the *i*-SFEM are lower than those of the *d*-FEM. The key difference is that the d-FEM simulation results in References [7,20] are pushed to exceed the experimental boundary range to achieve a match between the *i*-SFEM and experimental data. However, this study prioritizes the consistency between the *d*-FEM and the experiment, which serves as the foundation for uncertainty analysis, consequently leading to the results of the *i*-SFEM being lower than the experiments.

The underestimation tendency of the *i*-SFEM relative to the *d*-FEM can be attributed to the fact that the regions of weak material properties determine the damage initiation and propagation for in-plane tensile specimens due to stress concentration. Since the samples in the *i*-SFEM inevitably include data from the left side of the statistical distribution curve of material parameters (Figure 10b), this leads to premature damage initiation of structures. References [7,20] sacrificed the accuracy of the *d*-FEM to maintain the practical applicability of the *i*-SFEM in experimental observations. In this study, a refined mesh size was employed to achieve convergent prediction results, which implied a broader range of sample values. Thus, the deviation between the predicted result of the *i*-SFEM and the experiment was more pronounced. The situation reveals that the traditional independent sampling method was not suitable for predicting the stochastic mechanical properties of structures.

In comparison, the results from SFEMs constructed using dual-correlation sampling exhibited an increasing trend as the value of ***V*** increased. Notably, the mean value predicted by SFEM-II fell within the experimental range, revealing that the sampling method can effectively enhance the prediction accuracy for the stochastic mechanical responses of structures. To elucidate the underlying mechanism, Figure 10b presents the probability distributions of the samples of parameter *X_t_* for the three SFEMs. It can be observed that the samples from SFEM-II exhibit a distinctly steep distribution characteristic with a significant reduction in data variability due to the strong spatial autocorrelation. Moreover, there was a notable increase in the minimum value that dictates the structural strength, ultimately contributing to a marked improvement in the predicted results. This finding suggests that the spatial fluctuations of material properties within the composite structures are not only subtle but also exhibit significant spatial autocorrelation.

It is noted that a slightly lower mean value of SFEM-I was predicted compared to the result obtained from the *i*-SFEM, even though both the probability distributions were relatively similar. Analysis of the spatial distribution of parameter *X_t_* in Figure 10c–e reveals that samples acquired through dual-correlation sampling are influenced by surrounding material properties, exhibiting a weak zone clustering distribution. Specifically, samples with lower values aggregate to form mechanical property-deficient regions, causing initial damage to rapidly propagate along adjacent weak material zones. Consequently, SFEM-I predicts lower structural strength than the *i*-SFEM.

Additionally, it is observed that the error bars for structural strength predicted by the dual-correlation sampling method are significantly higher than those from the *i*-SFEM. Moreover, the corresponding error bars enlarge with the intensification of the degree of spatial autocorrelation. This is attributable to the fact that although the probability distribution of samples obtained by the *i*-SFEM exhibits the broadest range, the samples tend to approximate the statistical probability distribution of material parameters. The minimum values that determine structural performance in weak regions remain highly similar, resulting in prediction deviations not exceeding 6.5 MPa. It also demonstrates the insufficient capability of the independent sampling method in effectively reflecting the discreteness of structural strength performance. In contrast, samples considering spatial autocorrelation are influenced by initially randomly generated seeds, leading to pronounced fluctuations in both mean value and standard deviation, as illustrated in Figure 10d,e. Consequently, the predicted values exhibit remarkable randomness while remaining within the experimental deviation range. Specifically, the standard deviation of 17.0 MPa predicted by SFEM-II closely aligns with the experimental value of 21.3 MPa, which further corroborates the validity of the stochastic model.

**Figure 11:**
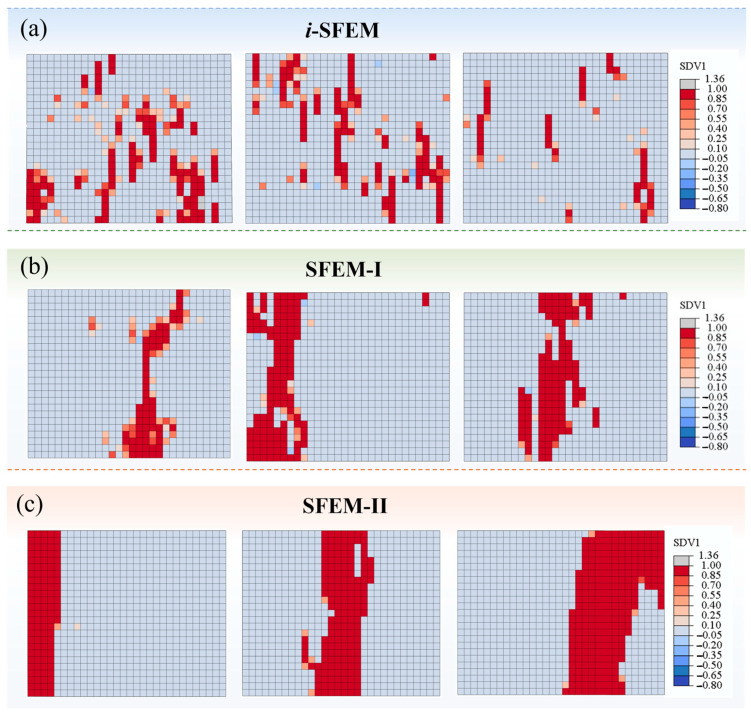
Comparison of typical damage contour maps for tensile specimens predicted by three SFEMs: (**a**) *i*-SFEM; (**b**) SFEM-I; (**c**) SFEM-II.

Furthermore, the discrete damage morphologies of tensile specimens predicted by the three SFEMs are presented in Figure 11. It can be observed that the results of the three models exhibit significant variability due to the gradual enhancement of spatial autocorrelation. A discrete and random point-like damage distribution is performed by the *i*-SFEM, which is consistent with the findings reported in Reference [7]. With the introduction and enhancement of spatial autocorrelation, the damage morphologies gradually transition to a narrow strip-like distribution and ultimately present a regular, wide-strip-like distribution. The variability coincides with the distribution of material properties under different spatial autocorrelation, as illustrated in Figure 10c–e, which thus elucidates the mechanism underlying the structural damage behaviors.

**Figure 12:**
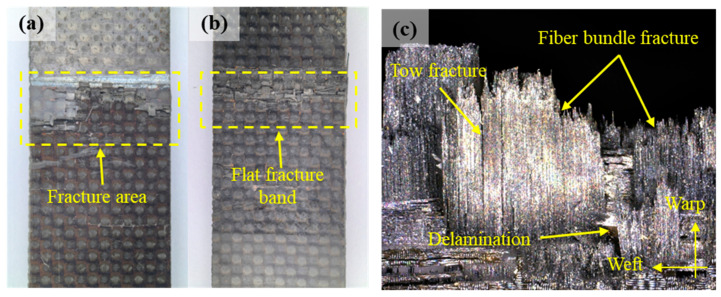
The optical fracture morphology of the tensile specimens: (**a**,**b**) typical macroscopic fractures captured by optical cameras; (**c**) fractures captured by the optical microscope.

For comparison, Figure 12 presents the optical fracture morphology for the tensile specimens. The fracture exhibits a relatively flat morphology, with the fracture plane oriented nearly perpendicular to the loading direction. Optical observation of the fracture surfaces reveals that the failure modes of fiber bundle breakage, splitting, and delamination at different interlaminar fracture sites collectively constitute the failure mechanism of the composite material structure.

The damage features predicted by the *i*-SFEM based on independent random sampling exhibit a discrete, randomly distributed point-like pattern, which contrasts with the actual fracture morphology observed experimentally. The prediction fails to effectively reflect the physical mechanisms governing the damage evolution of composite structures. In contrast, following the incorporation of spatial correlation, the predicted structural damage morphology exhibits regular patterns. The fracture zone predicted by SFEM-I appears relatively elongated and retains a degree of randomness, whereas that predicted by SFEM-II presents a broader and smoother profile. Since the brittle fracture bands predicted by both spatial models exhibit a degree of consistency with the experimental observation, the macroscopic comparative analysis indicates the accessibility of dual-correlation sampling in achieving reasonable predictive accuracy. To further clarify the differences between the two models, it is suggested to implement the sampling method in RVE models, which connects with the mesoscopic geometric morphology of structures.

Overall, compared to independent sampling methods, this study demonstrates the advantages of dual-correlation sampling in predicting the macroscopic fracture morphology of composite structures, which provides a more reliable tool for gaining deeper insights into the probabilistic load-bearing characteristics and discrete damage patterns.

## 4. Conclusions

This study focused on the prediction of the stochastic mechanical properties and damage behaviors of woven composite structures. A novel dual-correlation sampling approach based on multivariate random field theory was introduced. Through systematic numerical simulation and experimental validation, the main conclusions drawn are as follows:

(1) The dual-correlation sampling technique generated a “weakened zone clustering and strong zone continuous” performance distribution that closely resembles the real structures. Notably, the novel technique uniquely distinguished and coupled both the inter-specimen variability and intra-specimen heterogeneity of material property distributions, thereby reflecting the actual distribution of material properties in structural components.
(2) The mean value of the in-plane strengths of structures using the proposed method was 460.7 MPa, with a standard deviation of 17.0 MPa, which aligned well with the experimental results of 462.4 ± 21.3 MPa. In contrast, the independent sampling approach significantly underestimated both the mean strength and the standard deviation, leading to inaccurate probabilistic predictions.
(3) The dual-correlation sampling method effectively reproduced the spatial distribution characteristics of material properties, leading to more realistic predictions of damage morphology. As spatial correlation strengthened, damage patterns evolved from randomly distributed point-like failures to regular, band-like fracture zones, which aligned closely with experimental observations.

The proposed framework provided a robust and physically grounded approach for uncertainty propagation in composite structures, offering valuable guidance for reliability assessment and performance optimization in engineering applications. The next step of our work will focus on extending the approach to complex structural components to verify its applicability.

## Figures and Tables

**Table 1:** The probability distribution of the constituent material parameters.

Parameters	Mean Value	Standard Deviation	*CoV*.
Fiber longitudinal tensile strength $X_{ft}$/MPa	2500	28.24	1.11%
Fiber longitudinal compressive strength $X_{fc}$/MPa	2000	40.00	5.00%
Matrix tensile strength $X_{mt}$/MPa	60	2.75	4.59%
Matrix compressive strength $X_{mc}$/MPa	100	5.00	5.00%
Fiber volume fraction *V_f_*/%	0.5	0.025	5.00%

**Table 2:** Cross-correlation of in-plane strength parameters for plain woven composites obtained by correlation methods.

	Pearson Correlation Coefficient				Distance Correlation Coefficient		
	$X_{t}$	$X_{c}$	*S_c_*		$X_{t}$	$X_{c}$	*S_c_*
$X_{t}$	1	0.958	−0.284		1	0.916	0.099
$X_{c}$		1	−0.212			1	0.058
*S_c_*			1				1

**Table 3:** The distinction for two probability distributions of material parameters.

Symbol	Physical Meaning	Statistical Object	Distribution Source
$f_{i}$	Inter-specimen variability	Experimental macroscopic strength values of a batch of specimens	Multi-specimen experiments or RVE models
$f_{j}$	Intra-specimen spatial heterogeneity	Strength values of individual elements within a single specimen	Micro-area Raman spectroscopy or MRF sampling

**Table 4:** The FEMs for comparison with various parameter values of ***V***.

Model	Description	Model	Description
SFEM-I	***V*** =E	*i*-SFEM	Independent sampling, V→0
SFEM-II	***V*** =100·E	*d*-FEM	Deterministic FEM, V→∞

Where $E\in R^{3\times 3}$, with all elements being 1.0.

## Data Availability

The original contributions presented in this study are included in the article. Further inquiries can be directed to the corresponding authors.

## Appendix A

### Appendix A.1. The Construction of the Mesoscopic RVE Model

The variation of main material directions in the RVE model due to fiber bundle fluctuations needs to be defined. Given the immense number and irregularity of tetrahedral elements, it would be prohibitively labor-intensive to assign the main material direction for each element individually. To address this, the elements of fiber bundles are finely subdivided and organized into sets. By calculating the tangent angle for each set using Equation (2), the main material direction is uniformly defined for elements within the set, which, thereby, improves the modeling efficiency significantly. The transformation from the global main material datum ${\left[x_{i},y_{i}\right]}^{T}$ to the local material datum ${\left[x_{i}^{\prime },y_{i}^{\prime }\right]}^{T}\;$ is calculated by the following equation:

(A1) $$\left[\begin{array}{c} x_{i}\\ y_{i} \end{array}\right]=\left[\begin{array}{cc} \cos\phi & -\sin\phi \\ \sin\phi & \cos\phi \end{array}\right]\left[\begin{array}{c} {x_{i}}^{\prime }\\ {y_{i}}^{\prime } \end{array}\right]$$

By applying the transformation using Equation (A1), the local material orientation datum is defined along the fluctuated direction of both the weft and warp fiber bundle.

Periodic boundary conditions (PBCs) need to be applied in the RVE model. Generally, the PBCs for mesh model partitioned by regularly hexahedral elements can be uniformly expressed as [45]

(A2) $$u_{i}^{j+}-u_{i}^{j-}={\bar{\varepsilon }}_{ik}∆x_{k}^{j}$$

In the equation, ***u*** represents the displacement, where the superscripts *j+* and *j−* denote the master and slave surfaces of the RVE models, respectively, and subscript *i* and *k* are the nodes on the surfaces. $∆x_{k}^{j}$ is the spacing between the opposite surfaces, and $\;{\bar{\varepsilon }}_{ik}$ is the corresponding strain tensor. Since a one-to-one correspondence of nodes on the opposite surfaces cannot be guaranteed for the irregular tetrahedral elements in this study, an improved general periodic boundary condition (GPBC) is applied to the RVE model. For the point *P′* on the slave surface, which is the projection of node *P* on the master surface, its displacement can be described by interpolation using the displacements of the surrounding triangular nodes. Equation (A2) is modified as

(A3) $$u_{i}^{j+}-\left[\begin{array}{ccc} N_{1} & N_{2} & N_{3} \end{array}\right]\left[\begin{array}{c} u_{i}^{j-}\left(A\right)\\ u_{i}^{j-}\left(B\right)\\ u_{i}^{j-}\left(C\right) \end{array}\right]={\bar{\varepsilon }}_{ik}∆x_{k}^{j}$$

where *N_i_* represents the interpolation function, and the triangular isoparametric function is selected in the study. The modification ensures that the PBCs are no longer constrained by mesh quality but accessible to general cases.

### Appendix A.2. Constitutive Model of the Fiber Bundle and Matrix

A three-dimensional damage criterion is introduced to predict the damage initiation in yarns, as formulated in Table A1. In the expressions, $\tau_{ij}\;(i,j=1,2,3)$ represents the stress components along the *ij* directions. $X_{1t}$, $X_{2t}$, $X_{3t}$, $X_{1c}$, $X_{2c}$, $X_{3c}$,$S_{13}$, and $S_{23}$ are the strength parameters of the fiber bundle in various directions. Specifically, the shear shape factors α,β (0≤α,β≤1) are introduced into the failure criterion to characterize the influence of fluctuation effects on the damage behavior of fiber bundles under tensile–shear coupling. The values of the factors are both taken as 0.05 through the parameterized analysis of the mechanical properties of plain woven composites.

**Table A1:** Expressions for the three-dimensional failure criterion of fiber bundles.

Failure Mode	Expression
Longitudinal tensile failure	$e_{ft}={\left(\frac{\sigma_{11}}{X_{1t}}\right)}^{2}+\alpha \left(\frac{\tau_{12}^{2}+\tau_{13}^{2}}{S_{13}^{2}}\right)\geq 1$
Longitudinal compressive failure	$e_{fc}={\left(\frac{\sigma_{11}}{X_{1c}}\right)}^{2}+\alpha \left(\frac{\tau_{12}^{2}+\tau_{13}^{2}}{S_{13}^{2}}\right)\geq 1$
Transverse tensile failure	$e_{mt}={\left(\frac{\sigma_{22}}{X_{2t}}\right)}^{2}+\left[{\left(\frac{\tau_{12}}{S_{13}}\right)}^{2}+{\left(\frac{\tau_{23}}{S_{23}}\right)}^{2}\right]\geq 1$
Transverse compressive failure	$e_{mc}={\left(\frac{\sigma_{22}}{X_{2c}}\right)}^{2}+{\left(\frac{\tau_{12}}{S_{13}}\right)}^{2}+{\left(\frac{\tau_{23}}{S_{23}}\right)}^{2}\geq 1$
Out-of-plane tensile failure	$e_{zt}={\left(\frac{\sigma_{33}}{X_{3t}}\right)}^{2}+\beta \left[{\left(\frac{\tau_{13}}{S_{13}}\right)}^{2}+{\left(\frac{\tau_{23}}{S_{23}}\right)}^{2}\right]\geq 1$
Out-of-plane compressive failure	$e_{zc}={\left(\frac{\sigma_{33}}{X_{3c}}\right)}^{2}+\beta \left[{\left(\frac{\tau_{13}}{S_{13}}\right)}^{2}+{\left(\frac{\tau_{23}}{S_{23}}\right)}^{2}\right]\geq 1$

Stiffness degradation, followed by damage initiation, need to be considered in modeling the progressive failure behavior of fiber bundles. The damage evolution tensor $d_{i}$ for each failure mode is expressed by introducing an equivalent displacement as follows:

(A4) $$d_{i}=\frac{\delta_{i,eq}^{f}\left(\delta_{i,eq}-\delta_{i,eq}^{0}\right)}{\delta_{i,eq}\left(\delta_{i,eq}^{f}-\delta_{i,eq}^{0}\right)},\;i=ft,fc,mt,mc,zt,and\;zc$$

where $\delta_{i,eq}$ and $\sigma_{i,eq}$ represent the equivalent displacement and equivalent stress, respectively. $\delta_{i,eq}^{0}$ and $\delta_{i,eq}^{f}$ are the equivalent displacement and failure displacement calculated based on the fracture energy and equivalent stress, which control the rate of stiffness degradation.

The resin matrix exhibits typical elastic–plastic mechanical behavior, with yield behavior that is sensitive to hydrostatic pressure. The extended linear Drucker–Prager (D-P) criterion [46] is employed to describe the yield flow characteristic, with the expression given by

(A5) $$F=t-ptan\beta -d=0\;t=\frac{q}{2}\left[1+\frac{1}{k}-\left(1-\frac{1}{k}\right){\left(\frac{r}{q}\right)}^{3}\right]$$

where *p* is the hydrostatic pressure; *q* is the Mises equivalent stress; *r* is the third invariant of deviatoric stress; and β and *d* are the internal friction angle and cohesion, respectively. *k* represents the ratio of triaxial tensile strength to triaxial compressive strength, which governs the shape of the yield surface. In this study, it is set to 0.8.

The ductile criterion is implemented to describe the equivalent plastic strain $\varepsilon_{pl}^{0}$ at the onset of matrix damage with the consideration of different mechanical behavior under tensile and compressive loads. The $\varepsilon_{pl}^{0}$ is expressed as a function of the stress triaxiality *η*, whose values of 0, 1/3, and −1/3 correspond to shear, uniaxial tension, and uniaxial compression, respectively. The corresponding $\varepsilon_{pl}^{0}\;$ are set to 0.2, 0.02, and 0.2, respectively. Finally, the yield strain softening behavior during the process of damage evolution is defined using the dissipated energy, which is set to 5.0 J/m^2^.

The continuum damage mechanics’ constitutive relationship for fiber bundles is established using Matzenmiller’s theory [47]. A user-defined material model using the Abaqus/Explicit user interface VUMAT is developed to implement the above failure criterion and damage evolution. The element is deleted once the damage evolution tens $d_{ft}$ or $d_{fc}$ when the warp fiber bundle element reaches 1.0.

## References

[1] Zhang Y., Li Y., Luan X., Meng B., Liu J., Lu Y. (2025). Effects of Void Characteristics on the Mechanical Properties of Carbon Fiber Reinforced Polyetheretherketone Composites: Micromechanical Modeling and Analysis. Polymers.

[2] Yin Q.-Z., Bian J., Fan Y. (2025). A Numerical Study on Lightning Damages and Residual Strength of CFRP Laminates Considering Delamination Induced by Thermal Stress. Polymers.

[3] Zhou K., Wang Z., Gao Q., Yuan S., Tang J. (2023). Recent advances in uncertainty quantification in structural response characterization and system identification. Probabilistic Eng. Mech..

[4] Zhou X.Y., Qian S.Y., Wang N.W., Xiong W., Wu W.Q. (2022). A review on stochastic multiscale analysis for FRP composite structures. Compos. Struct..

[5] Zhao H., Zhou C. (2024). An imprecise multiscale uncertainty quantification framework for fiber reinforced composites. Probabilistic Eng. Mech..

[6] Wan A., Li D., Lu P. (2024). Three-scale modeling and probabilistic progressive damage analysis of woven composite laminates. Mech. Adv. Mater. Struct..

[7] Shah S., Lee J., Megat-Yusoff P., Hussain S.Z., Sharif T., Choudhry R. (2023). Multiscale damage modelling of notched and un-notched 3D woven composites with randomly distributed manufacturing defects. Compos. Struct..

[8] Generale A.P., Kalidindi S.R. (2023). Uncertainty quantification and propagation in the microstructure-sensitive prediction of the stress-strain response of woven ceramic matrix composites. Compos. Struct..

[9] Ali O., Bigaud D., Riahi H. (2018). Seismic performance of reinforced concrete frame structures strengthened with FRP laminates using a reliability-based advanced approach. Compos. Part B Eng..

[10] Zhou K., Enos R., Xu D., Zhang D., Tang J. (2022). Hierarchical multi-response Gaussian processes for uncertainty analysis with multi-scale composite manufacturing simulation. Comp. Mater. Sci..

[11] Zhu C., Zhu P., Liu Z. (2019). Uncertainty analysis of mechanical properties of plain woven carbon fiber reinforced composite via stochastic constitutive modeling. Compos. Struct..

[12] Zhang C.-Y., Wang Z., Fei C.-W., Yuan Z.-S., Wei J.-S., Tang W.-Z. (2019). Fuzzy Multi-SVR Learning Model for Reliability-Based Design Optimization of Turbine Blades. Materials.

[13] Zhao Y., Cheng X., Chen J., Xie K., Hu J. (2024). Cross-entropy based importance sampling for composite systems reliability evaluation with consideration of multivariate dependence. Int. J. Electr. Power Energy Syst..

[14] Balokas G., Czichon S., Rolfes R. (2018). Neural network assisted multiscale analysis for the elastic properties prediction of 3D braided composites under uncertainty. Compos. Struct..

[15] Ariyasinghe N., Herath S. (2024). Machine learning techniques for predictive modelling and uncertainty quantification of the mechanical properties of woven carbon fibre composites. Mater. Today Commun..

[16] Ciampaglia A. (2023). Data driven statistical method for the multiscale characterization and modelling of fiber reinforced composites. Compos. Struct..

[17] Han J., Wang R., Hu D., Bao J., Liu X., Guo X. (2022). A novel integrated model for 3D braided composites considering stochastic characteristics. Compos. Struct..

[18] Zhi J., Tay T.-E. (2018). Computational structural analysis of composites with spectral-based stochastic multi-scale method. Multiscale Multidiscip. Model. Exp. Des..

[19] Shi D., Teng X., Jing X., Lyu S., Yang X. (2020). A multi-scale stochastic model for damage analysis and performance dispersion study of a 2.5D fiber-reinforced ceramic matrix composites. Compos. Struct..

[20] Tao W., Zhu P., Xu C., Liu Z. (2020). Uncertainty quantification of mechanical properties for three-dimensional orthogonal woven composites. Part II: Multiscale simulation. Compos. Struct..

[21] Yang Q., Xu C., Cheng G., Meng S., Xie W. (2019). Uncertainty quantification method for mechanical behavior of C/SiC composite and its experimental validation. Compos. Struct..

[22] Sun T., Jiang R., Sun H., Liu D., Pan Z. (2023). Multiscale uncertainty propagation analysis and reliability optimization of the CFRP crossbeam of the twist beam axle. Int. J. Mech. Sci..

[23] Nastos C., Zarouchas D. (2022). Probabilistic failure analysis of quasi-isotropic CFRP structures utilizing the stochastic finite element and the Karhunen–Loève expansion methods. Compos. Part B Eng..

[24] Zhao Y., Vandepitte D., Lomov S.V. (2024). Fibre misalignments in the split-disk test represented by random fields. Compos. Part B Eng..

[25] Stefanou G., Savvas D., Gavallas P., Papaioannou I. (2022). The effect of random field parameter uncertainty on the response variability of composite structures. Comp. Part C Open Access.

[26] Arregui-Mena J.D., Worth R.N., Bodel W., März B., Li W., Campbell A.A., Cakmak E., Gallego N., Contescu C., Edmondson P.D. (2022). Multiscale characterization and comparison of historical and modern nuclear graphite grades. Mater. Char..

[27] Zhou K., Tang J. (2021). Uncertainty Quantification of Mode Shape Variation Utilizing Multi-Level Multi-Response Gaussian Process. J. Vib. Acoust..

[28] Bostanabad R., Liang B., Gao J., Liu W.K., Cao J., Zeng D., Su X., Xu H., Li Y., Chen W. (2018). Uncertainty quantification in multiscale simulation of woven fiber composites. Comput. Methods Appl. Mech. Eng..

[29] Jiang D., Nie W., Fei Q., Wu S. (2021). Free vibration analysis of composite panels considering correlations of spatially distributed uncertain parameters. Appl. Math. Model..

[30] Wang H., Wang B., Fu M., Fang G., Meng S. (2024). Spatial variability characterization and modelling of 2.5D woven SiO2f/SiO2 composites. Compos. Part A Appl. Sci. Manuf..

[31] (2017). Standard Test Method for Tensile Properties of Polymer Matrix Composite Materials.

[32] Chamis C.C. (1989). Mechanics of composite materials: Past, present, and future. J. Compos. Technol. Res..

[33] Chamis C.C. (1984). Simplified Composite Micromechanics Equations for Strength, Fracture Toughness, Impact Resistance and Environmental Effects. NASA Technical Memorandum. https://ntrs.nasa.gov/citations/19840059071.

[34] Doitrand A., Fagiano C., Irisarri F.-X., Hirsekorn M. (2015). Comparison between voxel and consistent meso-scale models of woven composites. Compos. Part A Appl. Sci. Manuf..

[35] Mileiko S.T. (2017). Fracture-toughness/notch-sensitivity correlation for metal- and ceramic-based fibrous composites. Compos. Part B Eng..

[36] Székely G.J., Rizzo M.L. (2013). Energy statistics: A class of statistics based on distances. J. Stat. Plan. Infer..

[37] Xiao S., Lu Z., Qin F. (2017). Estimation of the Generalized Sobol’s Sensitivity Index for Multivariate Output Model Using Unscented Transformation. J. Struct. Eng..

[38] Naik N.K., Ganesh V.K. (1996). Failure Behavior of Plain Weave Fabric Laminates under On-Axis Uniaxial Tensile Loading: I—Analytical Predictions. J. Compos. Mater..

[39] Shaw A., Sriramula S., Gosling P.D., Chryssanthopoulos M.K. (2010). A critical reliability evaluation of fibre reinforced composite materials based on probabilistic micro and macro-mechanical analysis. Compos. Part B Eng..

[40] Fedorov A.V., Gulyaeva Y.K. (2019). Strength statistics for porous alumina. Powder Technol..

[41] Tsuruta Y. (2024). Bias correction for kernel density estimation with spherical data. J. Multivar. Anal..

[42] Mazumder A., Zheng L., Jiao Y., Bullions T., Yu Y., Wang Y. (2024). Predictive modeling of 3D textile composites using realistic micromechanical representations. Compos. Part B Eng..

[43] Anderson T.W. (2003). An Introduction to Multivariate Statistical Analysis.

[44] Alshahrani H., Sebaey T.A. (2022). Bearing Properties of CFRP Composite Laminates Containing Spread-Tow Thin-Plies. Polymers.

[45] Xia Z., Zhou C., Yong Q., Wang X. (2006). On selection of repeated unit cell model and application of unified periodic boundary conditions in micro-mechanical analysis of composites. Int. J. Solids Struct..

[46] Zhou Y., Lu Z., Yang Z. (2013). Progressive damage analysis and strength prediction of 2D plain woven composites. Compos. Part B Eng..

[47] Yang L., Yan Y., Liu Y., Ran Z. (2012). Microscopic failure mechanisms of fiber-reinforced polymer composites under transverse tension and compression. Compos. Sci. Technol..

